# Assessment of Studies Evaluating Spinal Manipulative Therapy and Infectious Disease and Immune System Outcomes: A Systematic Review

**DOI:** 10.1001/jamanetworkopen.2021.5493

**Published:** 2021-04-13

**Authors:** Ngai Chow, Sheilah Hogg-Johnson, Silvano Mior, Carol Cancelliere, Stephen Injeyan, Julita Teodorczyk-Injeyan, J. David Cassidy, Anne Taylor-Vaisey, Pierre Côté

**Affiliations:** 1 Centre for Disability Prevention and Rehabilitation, Faculty of Health Sciences, Ontario Tech University, Oshawa, Ontario, Canada; 2 Canadian Memorial Chiropractic College, Toronto, Ontario, Canada; 3 Dalla Lana School of Public Health, University of Toronto, Toronto, Ontario, Canada

## Abstract

**Importance:**

Claims that spinal manipulative therapy (SMT) can improve immune function have increased substantially during the COVID-19 pandemic and may have contributed to the rapid spread of both accurate and inaccurate information (referred to as an infodemic by the World Health Organization).

**Objective:**

To identify, appraise, and synthesize the scientific literature on the efficacy and effectiveness of SMT in preventing the development of infectious disease or improving disease-specific outcomes in patients with infectious disease and to examine the association between SMT and selected immunological, endocrine, and other physiological biomarkers.

**Evidence Review:**

A literature search of MEDLINE, the Cumulative Index to Nursing and Allied Health Literature, the Index to Chiropractic Literature, the Cochrane Central Register of Controlled Trials, and Embase was conducted from inception to April 15, 2020. Randomized clinical trials and cohort studies were included. Eligible studies were critically appraised, and evidence with high and acceptable quality was synthesized using the Synthesis Without Meta-Analysis guideline.

**Findings:**

A total of 2593 records were retrieved; after exclusions, 50 full-text articles were screened, and 16 articles reporting the findings of 13 studies comprising 795 participants were critically appraised. The literature search found no clinical studies that investigated the efficacy or effectiveness of SMT in preventing the development of infectious disease or improving disease-specific outcomes among patients with infectious disease. Eight articles reporting the results of 6 high- and acceptable-quality RCTs comprising 529 participants investigated the effect of SMT on biomarkers. Spinal manipulative therapy was not associated with changes in lymphocyte levels or physiological markers among patients with low back pain or participants who were asymptomatic compared with sham manipulation, a lecture series, and venipuncture control groups. Spinal manipulative therapy was associated with short-term changes in selected immunological biomarkers among asymptomatic participants compared with sham manipulation, a lecture series, and venipuncture control groups.

**Conclusions and Relevance:**

In this systematic review of 13 studies, no clinical evidence was found to support or refute claims that SMT was efficacious or effective in changing immune system outcomes. Although there were limited preliminary data from basic scientific studies suggesting that SMT may be associated with short-term changes in immunological and endocrine biomarkers, the clinical relevance of these findings is unknown. Given the lack of evidence that SMT is associated with the prevention of infectious diseases or improvements in immune function, further studies should be completed before claims of efficacy or effectiveness are made.

## Key Points

**Question:**

Is spinal manipulative therapy associated with changes in the immune system?

**Findings:**

In this systematic review of 13 studies comprising 795 participants, no clinical studies investigated the efficacy or effectiveness of spinal manipulative therapy in preventing or improving disease-specific outcomes among patients with infectious disease. Preliminary laboratory experiments indicated that spinal manipulative therapy may, in the short term, be associated with levels of change in immunological biomarkers among asymptomatic participants.

**Meaning:**

These findings suggest that, given the limitations of the evidence, claims that spinal manipulative therapy is associated with changes in the immune system are premature and further clinical studies should be completed.

## Teaser

This systematic review investigates the association of spinal manipulative therapy with the prevention of infectious disease, improvement of disease-specific outcomes, and changes in immune system function.

## Introduction

At a time when the rapid spread of both accurate and inaccurate information (ie, infodemics, as referred to by the World Health Organization) has produced substantial concern for public health,^1,2^ claims that spinal manipulative therapy (SMT) can improve immune function have substantially increased with the onset of the COVID-19 pandemic, especially in Canada and the US.^3,4,5^ These claims were highlighted in a March 28, 2020, online report of the US-based International Chiropractors Association, which stated, “The observation that those who use chiropractic regularly and do not become ill with colds, flu, and other community shared illnesses is frequent within the profession and should not be ignored.”^6^ Such claims have a long history within the chiropractic profession.^7,8^ Proponents of these claims state that their position is informed by scientific evidence.^6^ However, the validity of these claims has been questioned.^9^

State chiropractic licensing boards in the US have provided limited and heterogenous guidance regarding chiropractic practice during the COVID-19 pandemic.^10^ In Canada, the College of Chiropractors of British Columbia, whose mission is to protect the public by regulating chiropractors to ensure safe, qualified, and ethical delivery of care,^11^ requested that we conduct an independent rapid review of the scientific literature to investigate the association of SMT with immunity. Therefore, we performed a systematic review of the literature to examine whether SMT was associated with efficacy and effectiveness in (1) preventing the development of infectious disease and (2) improving disease-specific health outcomes among patients with infectious disease. We also aimed to synthesize data from laboratory experiments to investigate the association between SMT and immunological, endocrine, and other physiological biomarkers.

## Methods

We conducted our review using the methodology recommended by the World Health Organization^12,13,14,15^ and the Preferred Reporting Items for Systematic Reviews and Meta-analyses (PRISMA) reporting guideline for systematic reviews.^16^ Ethics approval was not required, as this study was a systematic review. The review protocol was published in the Open Science Framework Registry on April 21, 2020^17^ (eMethods 1 in the Supplement).

We systematically searched MEDLINE, the Cumulative Index to Nursing and Allied Health Literature, the Index to Chiropractic Literature, the Cochrane Central Register of Controlled Trials, and Embase from inception to April 15, 2020. Search terms consisted of subject headings specific to each database (eg, medical subject headings in MEDLINE, such as *musculoskeletal manipulations*, *immunity*, and *communicable disease*) and free-text words relevant to our study objectives and design (search strategy and specific search terms are available in eMethods 2 in the Supplement). Randomized clinical trials (RCTs) and cohort studies were included if they were published in English, included participants who were healthy or symptomatic, examined SMT that was provided by any health care professional, compared SMT with no intervention or other interventions, and measured clinical outcomes or changes in the levels of immunological, endocrine, and other physiological biomarkers.

Data including study characteristics, participant demographic characteristics, intervention characteristics, and outcome data were extracted. The lead author (N.C.) critically appraised the internal validity of relevant articles using the Scottish Intercollegiate Guidelines Network criteria for RCTs and cohort studies.^18^ These appraisals were verified by the senior authors, which included epidemiologists (C.C., J.D.C., and P.C.), a biostatistician (S.H.J.), and general scientists (S.M., J.T., and S.I.). Disagreements regarding study quality were resolved through discussion. We categorized RCTs by study phases, as described by Campbell et al,^19^ and we synthesized the evidence from high- and acceptable-quality studies based on the Synthesis Without Meta-analysis guideline.^20^ We restricted the synthesis to studies with high and acceptable quality because those with low and unacceptable quality were more likely to yield biased estimates of effect sizes.^21,22,23,24,25^

## Results

The initial database search retrieved 2593 records. After excluding 598 duplicates, 1995 titles and abstracts were screened (Figure). The eligibility of a random sample of 200 titles and abstracts (10.0%) was then evaluated by 2 independent reviewers (N.C. and S.M.) to determine the interreviewer agreement of the screening process, which was found to be 98.5%. The full text of 50 articles was assessed to confirm eligibility.^26,27,28,29,30,31,32,33,34,35,36,37,38,39,40,41,42,43,44,45,46,47,48,49,50,51,52,53,54,55,56,57,58,59,60,61,62,63,64,65,66,67,68,69^ Of those, 16 articles^26,27,29,32,34,35,38,40,43,44,46,47,48,49,50,68^ reporting the results of 13 studies comprising 795 participants met the inclusion criteria and were critically appraised.

**Figure.**
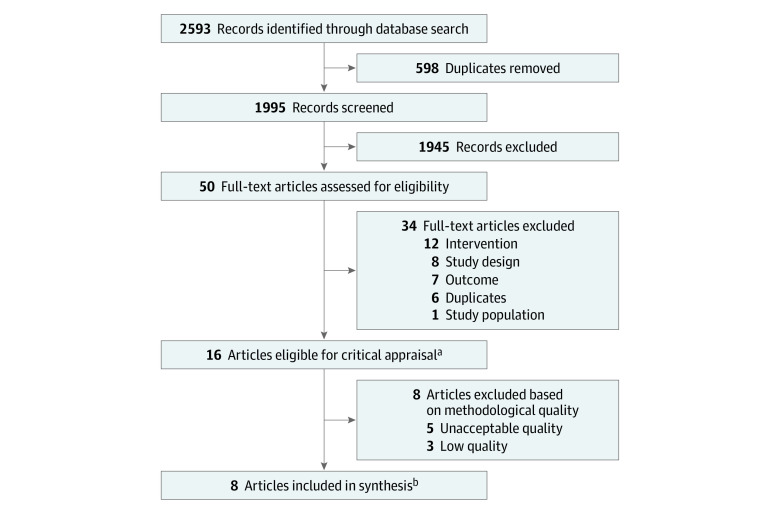
Flow Diagram of Study Selection ^a^The 16 eligible articles reported findings from 13 studies. ^b^Articles included in the synthesis reported findings from 6 randomized clinical trials, with 3 of those articles reporting the results of 1 randomized clinical trial.^26^

### Risk of Bias Within Studies

Of the 16 eligible articles, 1 article^50^ was rated as having high-quality evidence, and 7 articles^26,27,29,44,46,49,68^ were rated as having acceptable-quality evidence. Eight articles were rated as having low-quality^38,43^ or unacceptable-quality^32,34,35,40,47,48^ evidence; these articles were excluded from the evidence synthesis because of the high risk of bias. Studies with a high risk of bias had the following methodological limitations: (1) inadequate or unclear randomization (n = 5)^34,38,43,47,48^ and (2) inadequate or unclear allocation concealment (n = 3)^32,35,40^ (Table). We contacted the authors of 10 articles^32,34,35,38,40,43,47,48,49,50^ to inquire about their study methodology. Only 1 author responded and clarified that adequate methods were used for randomization, allocation concealment, and blinding.^49,50^ Therefore, 8 articles^26,27,29,44,46,49,50,68^ reporting the results of 6 high- or acceptable-quality RCTs comprising 529 participants were included in the evidence synthesis. These studies had various methodological limitations, including inadequate allocation concealment or blinding, dissimilar groups at baseline, and the use of outcomes with inadequate validity and reliability (Table).

**Table.** Risk of Bias

Source	Yes, no, or cannot be determined											Loss to follow-up	Quality of evidence
	Appropriate and clear research question	Adequate randomization	Adequate allocation concealment	Blinding of participants and investigators	Groups similar at start of study	Treatment is only difference between groups	Valid and reliable outcomes	Used intention-to-treat analysis	Results comparable between sites	Statistical analysis	Specimen collection		
Brennan et al,^50^ 1994	Y	Y	Y	Y	Y	Y	Y	N	NA	Y	Y	26.0% overall (unclear from which groups the participants withdrew)	High
Brennan et al,^49^ 1991	Y	Y	Y	Y	Y	Y	N	N	NA	Y	Y	9.5% SMT; 21.0% soft tissue; 7.9% sham	Acceptable
Mackawan et al,^27^ 2007	Y	Y	CBD	N	Y	Y	CBD	Y	NA	Y	Y	0% TTM; 0% joint mobilization	Acceptable
Puhl et al,^68^ 2012	Y	Y	N	N	CBD	Y	Y	NA	NA	Y	Y	5.3% SMT; 14.3% sham	Acceptable
Sampath et al,^44^ 2017	Y	Y	Y	CBD	N	Y	Y	Y	NA	Y	Y	0% TS SMT; 0% sham	Acceptable
Teodorczyk-Injeyan et al,^26^ 2006	Y	Y	N	Y	CBD	Y	Y	N	NA	CBD	Y	0% SMT; 0% sham; 0% VC	Acceptable
Teodorczyk-Injeyan et al,^29^ 2008	Y	Y	N	Y	CBD	Y	Y	N	NA	Y	Y	3.3% SMT; 8.0% sham; 11.0% VC	Acceptable
Teodorczyk-Injeyan et al,^46^ 2010	Y	Y	N	Y	CBD	Y	Y	N	NA	Y	Y	10.0% SMT; 0% sham; 18.5% VC	Acceptable
Molina-Ortega et al,^38^ 2014	Y	CBD	Y	CBD	CBD	Y	Y	Y	NA	Y	CBD	0% CS SMT; 0% TS SMT; 0% control	Low
Plaza-Manzano et al,^43^ 2014	Y	CBD	Y	N	N	CBD	Y	Y	NA	Y	Y	0% CS SMT; 0% TS SMT; 0% control	Low
Davison et al,^48^ 2003	Y	CBD	N	CBD	CBD	Y	CBD	Y	NA	N	Y	0% SMT; 0% sham	Unacceptable
Degenhardt et al,^35^ 2017	Y	Y	N	CBD	CBD	Y	Y	N	NA	Y	CBD	7.1% MT; 7.1% sham US; 0% no treatment	Unacceptable
Licciardone et al,^40^ 2012	Y	Y	CBD	CBD	N	CBD	Y	CBD	NA	Y	N	Not reported	Unacceptable
Licciardone et al,^32^ 2013	Y	Y	CBD	CBD	CBD	CBD	Y	CBD	NA	Y	N	Not reported	Unacceptable
Selano et al,^34^ 1994	N	CBD	N	CBD	CBD	CBD	Y	N	NA	N	N	55.0% overall (unclear from which groups the participants withdrew)	Unacceptable
Whelan et al,^47^ 2002	Y	CBD	N	CBD	CBD	CBD	Y	CBD	NA	N	N	Not reported	Unacceptable

Abbreviations: CS, cervical spine; CBD, cannot be determined; MT, manual treatment; NA, not applicable; US, ultrasound; SMT, spinal manipulative therapy; TS, thoracic spine; TTM, traditional Thai massage; VC, venipuncture control group.

### Study Characteristics

Among the 8 articles (6 RCTs)^26,27,29,44,46,49,50,68^ included in the evidence synthesis, 6 articles^26,29,46,49,50,68^ included SMT that was provided by chiropractors, and 2 articles^27,44^ included SMT that was provided by physiotherapists. Three RCTs^26,29,46,49,50^ evaluated the association between SMT and levels of immunological biomarkers (eTable 1 in the Supplement), and 3 RCTs^27,44,68^ examined the association between SMT and levels of endocrine and other physiological biomarkers (eTable 2 in the Supplement). Four RCTs^26,29,44,46,49,68^ included healthy and/or asymptomatic adults, while 2 RCTs^27,50^ included adult patients with low back pain. No studies reported on the incidence of infections, and no studies included children or patients with an infectious disease.

### Evidence Synthesis

We did not identify any eligible RCTs or cohort studies that investigated the association between SMT and efficacy, effectiveness, or surveillance in the prevention of infectious disease. We also did not find any eligible RCTs or cohort studies that investigated the association between SMT and efficacy, effectiveness, or surveillance in the improvement of disease-specific outcomes among patients with infectious disease.

Spinal manipulative therapy was associated with immediate changes in the levels of selected immunological biomarkers (polymorphonuclear neutrophils,^49^ monocytes,^49^ tumor necrosis factor α,^26^ interleuken 1β,^26^ interleuken 2,^29^ immunoglobulin G,^46^ and immunoglobulin M^46^) in asymptomatic participants compared with sham manipulation and a lecture series^49^ and with venipuncture control groups.^26,29,46^ The duration of these changes and their physiological or clinical significance is unknown. However, SMT was not associated with changes in lymphocyte levels^46,50^ among patients with low back pain^50^ or participants who were asymptomatic^46^ compared with sham manipulation and a lecture series^50^ and with sham manipulation and venipuncture control groups (eTable 1 in the Supplement).^46^ With the exception of 1 study, which was designed solely to investigate associations between SMT and changes in lymphocyte subpopulations,^50^ the levels of biomarkers of interest were assessed in vitro^26,29,46,49^ (eTable 1 in the Supplement).

In all studies with the exception of one,^44^ SMT was not associated with levels of selected physiological markers (substance P,^26,27,49^ testosterone,^44^ testosterone to cortisol ratio,^44^ oxyhemoglobin,^44^ heart rate variability,^44^ norepinephrine,^68^ or epinephrine^68^), in the immediate term among patients with chronic low back pain^27^ or participants who were asymptomatic^44^ (eTable 1 and eTable 2 in the Supplement). However, 1 study^44^ reported that SMT was associated with changes in the level of salivary cortisol in the immediate term among asymptomatic participants compared with sham SMT (eTable 2 in the Supplement). The physiological or clinical significance of these changes and their duration is unknown.

## Discussion

In this systematic review, no evidence from acceptable- or high-quality RCTs was found to support or refute the efficacy or effectiveness of SMT to prevent the development of infectious disease or to improve disease-specific outcomes among patients with infectious disease through its consequences for the immune system. Although 8 high- or acceptable-quality articles were identified that suggested SMT may be associated with immediate changes in immunological and endocrine biomarkers, these findings were preliminary and were mostly based on in vitro observations that appeared to be dependent on study methodology. Furthermore, the studies were conducted among asymptomatic participants or patients with low back pain, and their clinical relevance is unknown. It is important to note that all of the studies included in this systematic review were phase 0 (exploratory) studies, including those with a high risk of bias that were not included in the synthesis and therefore could not be used to assess the efficacy and effectiveness of SMT in preventing the development of infectious disease or improving disease-specific outcomes in patients with infectious disease.

### Implications and Future Research

The findings from this review were based on phase 0 (exploratory) studies that included healthy participants or patients with low back pain but did not include patients with an infectious disease. None of the studies included in the review were phase 2 (biologic activity) studies, which would have established proof of concept that an intervention had any biologic activity. Although some of the studies in the present review appeared to indicate an association between SMT and selected immunological parameters, the clinical implications of SMT for the immune system are unknown.

Any intervention must be properly evaluated in clinical trials, including phase 2 (biologic activity) and phase 3 (efficacy or effectiveness) studies, before widespread use. The findings from several phase 0 (exploratory) studies suggested that there was a possible association between SMT and selected biomarkers. However, no clear and consistent associations across studies were identified. Moreover, none of the included studies considered the implications of SMT for immunity but instead explored the association between SMT and selected biomarkers associated with the human inflammatory response. If this area of research is to be prioritized, then the exploratory nature of our findings and the current available evidence-based interventions for the treatment of infectious diseases should be considered, and appropriately designed clinical trials should be conducted. However, given the current lack of clear and consistent data regarding the association between SMT and immune markers, it would be premature to conduct RCTs without high-quality evidence from future phase 0 clinical trials.

### Strengths and Limitations

This study has several strengths. These strengths include adherence to the PRISMA checklist,^16^ establishment of a protocol before review completion and registration with the Open Science Framework Registry, formulation of a clear research question, use of a robust literature search strategy of 5 databases that was reviewed by ^2^ librarians, screening of interrater reliability comparison, critical appraisal of eligible studies, and inclusion of a review process that was conducted by senior scientists at each step of the rapid review. We also provided a full electronic search strategy, including limits used, for at least 1 database so that the search can be replicated.

This study also has limitations. First, we only included studies published in English and in peer-reviewed journals, which has the potential to introduce publication bias; however, most studies are published in English,^70^ and the exclusion of articles written in a language other than English would not be likely to produce bias, given that most clinical trials are published in the English-language literature.^71,72,73,74,75^ Furthermore, we consulted content experts (S.I., J.T.I., and J.D.C.) regarding their knowledge of other studies in the field to minimize publication bias. Second, screening, critical appraisal, and data extraction were conducted by 1 investigator rather than 2. However, we implemented a structured quality assurance methodology to minimize errors in the screening and selection of articles and data extraction.

## Conclusions

No clinical evidence from high- or acceptable-quality RCTs was found to support or refute claims that SMT is efficacious or effective in preventing or improving infectious diseases. We found limited exploratory evidence from primarily in vitro studies that SMT may be associated with immediate changes in immunological and endocrine biomarkers among healthy participants or patients with low back pain. However, the clinical relevance of these findings is unknown, particularly among patients with infectious disease. Given the lack of evidence that SMT prevents infectious diseases or improves immune function, further studies are warranted before claims of efficacy or effectiveness can be made.

## Notes

Supplement. **eMethods 1.** Protocol and Registration, Eligibility Criteria, Information Sources, Study Selection, Risk of Bias in Individual Studies, Data Extraction, Data Items, Statistical Analysis, and Evidence Synthesis **eMethods 2.** Search Strategies **eTable 1.** Evidence Table for Immune Markers **eTable 2.** Evidence Table for Endocrine and Other Physiological Markers **eReferences**
