# Supplementary material for: Assessment of Studies Evaluating Spinal Manipulative Therapy and Infectious Disease and Immune System Outcomes: A Systematic Review
Source: JAMA Netw Open. 2021 Apr 13;4(4):e215493. doi: 10.1001/jamanetworkopen.2021.5493 (PMC8044731; doi:10.1001/jamanetworkopen.2021.5493)
Supplement: Supplement. — eMethods 1. Protocol and Registration, Eligibility Criteria, Information Sources, Study Selection, Risk of Bias in Individual Studies, Data Extraction, Data Items, Statistical Analysis, and Evidence Synthesis eMethods 2. Search Strategies eTable 1. Evidence Table for Immune Markers eTable 2. Evidence Table for Endocrine and Other Physiological Markers eReferences [file jamanetwopen-e215493-s001.pdf]

Supplementary Online Content

Chow N, Hogg-Johnson S, Mior S, et al. Assessment of studies evaluating spinal manipulative therapy and infectious disease and immune system outcomes: a systematic review. *JAMA Netw Open*. 2021;4(4):e215493. doi:10.1001/jamanetworkopen.2021.5493

eMethods 1. Protocol and Registration, Eligibility Criteria, Information Sources, Study Selection, Risk of Bias in Individual Studies, Data Extraction, Data Items, Statistical Analysis, and Evidence Synthesis

eMethods 2. Search Strategies

eTable 1. Evidence Table for Immune Markers

eTable 2. Evidence Table for Endocrine and Other Physiological Markers

eReferences

This supplementary material has been provided by the authors to give readers additional information about their work.

© 2021 Chow N et al. *JAMA Network Open*.

eMethods 1. Protocol and Registration, Eligibility Criteria, Information Sources, Study Selection, Risk of Bias in Individual Studies, Data Extraction, Data Items, Statistical Analysis, and Evidence Synthesis

We conducted a systematic review of the literature. Rapid reviews are used by health decision-makers (clinicians, patients, managers, and policy makers) who need timely access to health information to plan, develop and implement health care and policies.[9-12] We used methodology recommended by the World Health Organization, previously used by our group,[13,14] to answer our questions.

Protocol and Registration

We reported our review according to the Preferred Reporting Items for Systematic Reviews and Meta-Analysis (PRISMA) checklist.[15] The review protocol was submitted to the Open Science Framework Registry on April 21, 2020 (https://osf.io/ux7wc).

Eligibility Criteria

Participants

We included studies of healthy and symptomatic participants.

Interventions

SMT includes spinal manipulation and spinal mobilization provided by any type of provider. Spinal manipulation includes techniques incorporating a high-velocity, low amplitude impulse or thrust (HVLA) applied at or near the end of a joint's passive range of motion.[16] Spinal mobilization includes techniques incorporating low-velocity and varying amplitude oscillatory movements within a joint's passive range of motion.[16-18] Spinal manipulation and mobilization involve manual, mechanically- or instrument-assisted procedures.

Comparators

We considered placebo, sham therapies, wait listing, usual care, no interventions, medication and other therapies.

Outcomes

We investigated clinical outcomes and changes in levels of immunological, endocrine and other physiological biomarkers. Clinical outcomes included incidence of infection or infectious disease, or changes in their disease-specific outcomes (e.g. number and severity of symptoms or faster recovery from illness). A biomarker is a "characteristic that is objectively measured and evaluated as an indicator of normal biological processes, pathogenic processes, or pharmacologic responses to a therapeutic intervention."[19] Biomarkers encompass a multitude of physiological indicators including molecules in the immune system such as cytokines, and chemokines, as well as endocrine biomarkers such as cortisol, testosterone, catecholamines and others.

Study Designs

Eligible study designs were randomized controlled trials (RCTs) and cohort studies. All other study designs were excluded.

Information Sources

The search strategy was developed in consultation with a health sciences librarian, and a second librarian reviewed the strategy to ensure accuracy. We systematically searched five databases that index the manual therapy literature published by researchers from various health professions from inception to April 15, 2020: MEDLINE (U.S. National Library of Medicine, through Ovid Technologies Inc.), Cumulative Index to Nursing and Allied Health Literature (CINAHL, through EBSCOhost), Index to Chiropractic Literature (ICL, Chiropractic Library Collaboration), Cochrane Central Register of Controlled Trial and Embase (both through Ovid Technologies Inc). Search terms consisted of subject headings specific to each database (e.g. MeSH in MEDLINE) and free text words relevant to our objectives and study design (see Appendix 1). We restricted our search to papers published in English in peer-reviewed journals.

Study Selection

A two-phase screening process was used to identify eligible studies. In phase one screening, we reviewed titles and abstracts and classified articles as possibly relevant or irrelevant. During phase two screening, the full text of possibly relevant articles was reviewed for final determination of eligibility. A trained reviewer (NC) conducted all of the

© 2021 Chow N et al. *JAMA Network Open*.

screenings. Prior to phase one screening, the quality of screening by NC was established by randomly selecting ten percent of all eligible articles, which were screened independently by a second experienced reviewer (SM). A 95% level of agreement was required between the two reviewers before moving to full screening. Once the 95% agreement was achieved, one reviewer (NC) completed phase one screening. For phase two screening, all potentially eligible articles were screened by rotating pairs of experienced reviewers (PC, CC, SM, JDC, SI, JTI).

Risk of Bias in Individual Studies

The lead author (NC) critically appraised the internal validity of relevant articles using the Scottish Intercollegiate Guidelines Network (SIGN) criteria for RCTs and cohort studies.[20] A quality control step in the critical appraisal of studies was included, wherein the investigator assessing the risk of bias (NC) presented a summary of the critical appraisals to seven experienced reviewers (PC, SHJ, CC, SM, JDC, SI, JTI) who validated the outcome of the appraisals. Disagreements regarding the internal validity of papers were resolved through discussion. The lead author created risk of bias tables for all eligible studies, which was validated by the other reviewers (PC, SHJ, CC, SM, JDC, SI, JTI).

Data Extraction

The lead author (NC) extracted data from high and acceptable quality (low risk of bias) studies and built evidence tables stratified by study objective and nature of the outcome. Data extraction was validated by the other reviewers (PC, SHJ, CC, SM, JDC, SI, JTI). We contacted the study authors when clarification or additional information was necessary to populate the evidence tables. Evidence tables summarized the pertinent information and were used to create summary statements describing the state of the evidence.

Data Items

Information extracted from each study included study characteristics, (e.g. author, year and country of publication, study design, sample size, length of follow up), participant characteristics (e.g., age, and body region treated), intervention characteristics (e.g., description of interventions and comparison groups), and outcome data (e.g., incidence of infection or infectious disease, disease-specific outcomes, changes in the levels of immunological, endocrine and other physiological biomarkers).

Classification of Clinical Trial Phases

We categorized RCTs into the different phases of clinical trials as described by Campbell et al.[21] The purpose of a phase 0 (exploratory) study is to gather preliminary data on whether the intervention behaves as expected in humans based on preclinical studies, and involves limited human exposure (10-15 healthy volunteers) to the intervention, with no therapeutic or diagnostic goals.[21] A phase 1 (safety) study aims to determine safety of the intervention and typically involves 20-80 healthy volunteers.[21] A phase 2 (biologic activity) study aims to establish proof of concept that the intervention has biologic activity, usually involving 100-300 patients with specific diseases.[21] A phase 3 (efficacy) study aims to confirm the efficacy and effectiveness of the intervention, monitor side effects, and frequently involves 300-3000 patients with specific diseases.[21] A phase 4 (post-marketing/confirmatory) study aims to provide surveillance and additional information on the intervention's risks, benefits and best use after the intervention is approved for human use, and involves several thousand patients treated with the intervention.[21]

Statistical Analysis

We reported or computed (when data were available), the incidence and 95% confidence intervals (CI) of infection or infectious disease, and mean difference and 95% CI in change in the level of biomarkers. We reported or calculated the pre-post difference in means or mean differences between groups based on data derived from regression models. Incidence was measured by calculating the number of new cases of infection or infectious disease in a group divided by the total number of participants in the same group. Confidence intervals were calculated using incidence of infection or infectious disease in each group, total number of participants in each group, and $\alpha = 0.05$.

Evidence Synthesis

We synthesized the evidence from high and acceptable quality studies according to the Synthesis without Meta-Analysis (SWiM) Guideline,[22] study objective, and type of biomarker. We restricted our synthesis to studies with high or acceptable quality because low/unacceptable quality studies are more likely to yield biased estimates of effect sizes.[23-27]

© 2021 Chow N et al. *JAMA Network Open*.

eMethods 2. Search Strategies

CCBC_RR3_Immunesystem_MEDLINE
Search run April 16, 2020 in Ovid MEDLINE: Epub Ahead of Print, In-Process & Other Non-Indexed Citations, Ovid MEDLINE® Daily and Ovid MEDLINE® 1946-Present; 634 results

1	Musculoskeletal Manipulations/	1732
2	Manipulation, Spinal/	1568
3	Manipulation, Chiropractic/	985
4	Manipulation, Orthopedic/	3891
5	Manipulation, Osteopathic/	1007
6	(Activator adj (method or tool*)).ab,ti.	7
7	(adjust* adj3 (chiropract* or spinal or lumbar or cervical or thoracic or instrument* or tool* or electric)).ab,ti.	1750
8	(flexion-distraction or flexion distraction).ab,ti.	257
9	(HVLA or high velocity low amplitude).ab,ti.	268
10	(manipulat* adj3 (chiropract* or naprapath* or osteopath* or orthopedic* or orthopaedic*)).ab,ti.	1502
11	(manipulat* adj3 (spinal or spine or low* back or joint* or lumbar or neck or therap* or thoracic or cervical or intervention* or MSK or manage* or musculoskeletal or treat* or vertebr*)).ab,ti.	9359
12	(instrument* adj3 (manual or mobili?at* or manipulat*)).ab,ti.	1111
13	(manipulat* adj3 (physiotherap* or physical therap*)).ab,ti.	264
14	(mobili?at* adj3 (chiropract* or naprapath* or osteopath* or orthopedic* or orthopaedic*)).ab,ti.	29
15	(mobili?at* adj3 (spinal or spine or low* back or joint* or lumbar or neck or thoracic or cervical or MSK or musculoskeletal or vertebr* or therap* or treat* or intervention* or manage*)).ab,ti.	2720
16	((manipulat* or mobili?at*) adj4 instrument*).ab,ti.	767
17	(manual adj3 (therap* or treat* or intervention* or manag*)).ab,ti.	5252
18	or/1-17 [**intervention]	25062
19	Case-Control Studies/	281252
20	Clinical Trials as Topic/	190776
21	clinical trial.pt.	522192
22	Cohort Studies/	259025
23	controlled clinical trial.pt.	93621
24	Controlled Clinical Trials as Topic/	5500
25	Cross-Over Studies/	47515
26	Double Blind Method/	157136
27	Epidemiologic Studies/	8272
28	Follow-Up Studies/	638093
29	Longitudinal Studies/	132993
30	Observational Study.pt.	77739
31	Prospective Studies/	534985
32	Random Allocation/	102602
33	randomized controlled trial.pt.	503984

© 2021 Chow N et al. *JAMA Network Open*.

34	exp Randomized Controlled Trial/	504766
35	Randomized Controlled Trials as Topic/	132074
36	Retrospective Studies/	811803
37	Single Blind Method/	28369
38	((crossover or cross-over) adj3 (study or studies or trial*)).ab,ti.	44437
39	((followup or follow-up) adj3 (stud* or design* or analysis)).ab,kf,ti.	80973
40	"cohort*".ab,ti.	574314
41	(control* adj3 (group* or trial*)).ab,ti.	765649
42	(longitudinal* adj3 (stud* or design* or analysis)).ab,ti.	116244
43	(observational adj3 (stud* or design* or analysis)).ab,ti.	141167
44	(prospective adj3 (stud* or design* or analysis)).ab,ti.	376632
45	(random* adj5 (allocat* or assign* or control* or clinical or patient* or sample* or trial*)).ab,ti.	657292
46	(retrospective adj3 (stud* or design*)).ab,ti.	298953
47	((singl* or doubl* or treb* or tripl*) adj3 (blind* or mask*)).ab,ti.	172585
48	or/19-47 [**study designs]	4133448
49	"Allergy and Immunology"/	6771
50	Anti-Allergic Agents/	8013
51	exp Antibodies/	814756
52	Biomarkers/	271257
53	exp CD4 Lymphocyte Count/	25969
54	exp Chemokines, CC/	24003
55	exp Communicable Diseases/	35019
56	Cytokines/	145261
57	exp Endocrine System/	442746
58	exp Immune System/	1141233
59	exp Immunity/	336653
60	exp Immunoglobulins/	879375
61	exp Infections/	2568934
62	Inflammation/	154033
63	Inflammation Mediators/	32052
64	exp Interleukin-1/	58422
65	Luminescent Measurements/	27802
66	Monocytes/	57030
67	Neutrophils/	86212
68	Radioimmunoassay/	64573
69	Respiratory Burst/	5093
70	exp Selectins/	14574
71	Th1 Cells/	17943
72	Th2 Cells/	16723

© 2021 Chow N et al. *JAMA Network Open*.

73	exp Tumor Necrosis Factors/	151654
74	exp Viruses/	770978
75	allergy.ab,ti.	64878
76	(anti-allerg* or antiallerg*).ab,ti.	5310
77	"antibod*".ab,ti.	842302
78	biomarker*.ab,ti.	249353
79	CD4.ab,ti.	149616
80	"chemokine*".ab,ti.	67844
81	"cytokine*".ab,ti.	354029
82	(disease* adj4 (communicable or infectious)).ab,ti.	99375
83	endocrine.ab,ti.	120124
84	(immune or immunity).ab,ti.	711778
85	"immunoglobulin*".ab,ti.	152245
86	immunology.ab,ti.	22543
87	"infection*".ab,ti.	1357011
88	"inflammation*".ab,ti.	420522
89	(Interleukin-1 or Interleukin-2).ab,ti.	63128
90	"luminescent measurement*".ab,ti.	27
91	monocytes.ab,ti.	69780
92	neutrophils.ab,ti.	78121
93	radioimmunoassay.ab,ti.	49274
94	respiratory burst.ab,ti.	5826
95	selectins.ab,ti.	2081
96	substance P.ab,ti.	22186
97	(Th1 or Th2).ab,ti.	52229
98	(TNFalpha or TNF-alpha).ab,ti.	149691
99	Tumor Necrosis Factor-alpha.ab,ti.	76702
100	or/49-99 [**immune_system]	6624939
101	18 and 48 and 100	719
102	limit 101 to english language	634

Index to Chiropractic Literature
Search run April 16, 2020. Truncated text words for all MEDLINE search lines searched, as well as known authors. Case reports and conference abstracts removed. 30 articles downloaded.

Description	Articles	Date	
S1	All Fields:manipulat* OR All Fields:activator OR All Fields:adjustment* OR All Fields:flexion OR All Fields:HVLA OR All Fields:instrument* OR All Fields:manual, Peer Review only	3799	2020-04-16 15:15:35
S2	All Fields:allergy OR All Fields:antibod* OR All Fields:biomarker* OR All Fields:CD4 OR All Fields:Chemokine* OR Subject:\"Communicable Diseases\" OR All Fields:cytokine* OR All Fields:endocrine OR All Fields:immune OR All Fields:immunity OR	28	2020-04-16 15:19:44

© 2021 Chow N et al. *JAMA Network Open*.

	All Fields:Immunoglobulin* OR All Fields:Interleukin OR All Fields:Monocyte* OR All Fields:TH2 AND All Fields:infection* OR All Fields:infectious, Peer Review only		
S3	All Fields:manipulat* OR All Fields:activator OR All Fields:adjustment* OR All Fields:flexion OR All Fields:HVLA OR All Fields:instrument* OR All Fields:manual, Peer Review only AND All Fields:allergy OR All Fields:antibod* OR All Fields:biomarker* OR All Fields:CD4 OR All Fields:Chemokine* OR Subject:\"Communicable Diseases\" OR All Fields:cytokine* OR All Fields:endocrine OR All Fields:immune OR All Fields:immunity OR All Fields:Immunoglobulin* OR All Fields:Interleukin OR All Fields:Monocyte* OR All Fields:TH2 AND All Fields:infection* OR All Fields:infectious, Peer Review only	10	2020-04-16 15:20:12
S4	All Fields:immune OR All Fields:autoimmune OR All Fields:neuroimmune OR All Fields:immun* OR Author:\"Injeyan HS\" OR Author:\"Teodorczyk-Injeyan JA\", Peer Review only	148	2020-04-16 15:24:57
S5	All Fields:manipulat* OR All Fields:activator OR All Fields:adjustment* OR All Fields:flexion OR All Fields:HVLA OR All Fields:instrument* OR All Fields:manual, Peer Review only AND All Fields:allergy OR All Fields:antibod* OR All Fields:biomarker* OR All Fields:CD4 OR All Fields:Chemokine* OR Subject:\"Communicable Diseases\" OR All Fields:cytokine* OR All Fields:endocrine OR All Fields:immune OR All Fields:immunity OR All Fields:Immunoglobulin* OR All Fields:Interleukin OR All Fields:Monocyte* OR All Fields:TH2 AND All Fields:infection* OR All Fields:infectious, Peer Review only OR All Fields:immune OR All Fields:autoimmune OR All Fields:neuroimmune OR All Fields:immun* OR Author:\"Injeyan HS\" OR Author:\"Teodorczyk-Injeyan JA\", Peer Review only	152	2020-04-16 15:25:14
S6	All Fields:manipulat* OR All Fields:activator OR All Fields:adjustment* OR All Fields:flexion OR All Fields:HVLA OR All Fields:instrument* OR All Fields:manual, Peer Review only AND All Fields:manipulat* OR All Fields:activator OR All Fields:adjustment* OR All Fields:flexion OR All Fields:HVLA OR All Fields:instrument* OR All Fields:manual, Peer Review only AND All Fields:allergy OR All Fields:antibod* OR All Fields:biomarker* OR All Fields:CD4 OR All Fields:Chemokine* OR Subject:\"Communicable Diseases\" OR All Fields:cytokine* OR All Fields:endocrine OR All Fields:immune OR All Fields:immunity OR All Fields:Immunoglobulin* OR All Fields:Interleukin OR All Fields:Monocyte* OR All Fields:TH2 AND All Fields:infection* OR All Fields:infectious, Peer Review only OR All Fields:immune OR All Fields:autoimmune OR All Fields:neuroimmune OR All Fields:immun* OR Author:\"Injeyan HS\" OR Author:\"Teodorczyk-Injeyan JA\", Peer Review only	55	2020-04-16 15:25:27
S7	Author:\\\"Campbell CJ\\\"	1	2020-04-16 15:30:43
S8	Author:\\\"Pero RW\\\"	1	2020-04-16 15:31:08
S9	Author:\"Selano J\" OR Author:\"Selano JL\", Peer Review only	1	2020-04-16 15:32:03

© 2021 Chow N et al. *JAMA Network Open*.

Embase
CCBC_RR3 _Immunesystem_Embase
Search run April 16, 2020 in Embase Classic+Embase 1947 to 2020 April 16: 1259 results

1	musculoskeletal manipulation/	306
2	spine manipulation/	542
3	chiropractic manipulation/	234
4	orthopedic manipulation/	2363
5	osteopathic manipulation/	301
6	manipulative medicine/	10934
7	(Activator adj (method or tool*)).ab,ti.	58
8	(adjust* adj3 (chiropract* or spinal or lumbar or cervical or thoracic or instrument* or tool* or electric)).ab,ti.	2300
9	(flexion-distraction or flexion distraction).ab,ti.	285
10	(HVLA or high velocity low amplitude).ab,ti.	279
11	(manipulat* adj3 (chiropract* or naprapath* or osteopath* or orthopedic* or orthopaedic*)).ab,ti.	1896
12	(manipulat* adj3 (spinal or spine or low* back or joint* or lumbar or neck or therap* or thoracic or cervical or intervention* or MSK or manage* or musculoskeletal or treat* or vertebr*)).ab,ti.	12195
13	(instrument* adj3 (manual or mobili?at* or manipulat*)).ab,ti.	1514
14	(manipulat* adj3 (physiotherap* or physical therap*)).ab,ti.	349
15	(mobili?at* adj3 (chiropract* or naprapath* or osteopath* or orthopedic* or orthopaedic*)).ab,ti.	38
16	(mobili?at* adj3 (spinal or spine or low* back or joint* or lumbar or neck or thoracic or cervical or MSK or musculoskeletal or vertebr* or therap* or treat* or intervention* or manage*)).ab,ti.	4032
17	(manual adj3 (therap* or treat* or intervention* or manag*)).ab,ti.	7786
18	((therap* or treat* or intervention* or manag*) adj3 (manual or manipulat* or mobili?at* or MSK or musculoskeletal)).ab,ti.	23385
19	or/1-18 [** spinal_manipulation]	39737
20	case control study/	154150
21	"clinical trial (topic)"/	107102
22	clinical trial/	993257
23	cohort analysis/	567069
24	"controlled clinical trial (topic)"/	10675
25	controlled study/	7355449
26	controlled clinical trial/	464348
27	double blind procedure/	173984
28	follow up/	1566467
29	longitudinal study/	138855
30	observational study/	193758
31	prospective study/	595453
32	random sample/	12945

© 2021 Chow N et al. *JAMA Network Open*.

33	randomized controlled trial/	601440
34	"randomized controlled trial (topic)"/	177191
35	retrospective study/	906072
36	single blind procedure/	38585
37	((crossover or cross-over) adj3 (study or studies or trial*)).ab,ti.	58815
38	[((followup or follow-up) adj3 (stud* or design* or analysis)).ab,kf,ti.]	0
39	"cohort*".ab,ti.	976377
40	(control* adj3 (group* or trial*)).ab,ti.	1092420
41	(longitudinal* adj3 (stud* or design* or analysis)).ab,ti.	155879
42	(observational adj3 (stud* or design* or analysis)).ab,ti.	222054
43	(prospective adj3 (stud* or design* or analysis)).ab,ti.	558139
44	(random* adj5 (allocat* or assign* or control* or clinical or patient* or sample* or trial*)).ab,ti.	916947
45	(retrospective adj3 (stud* or design*)).ab,ti.	468052
46	((singl* or doubl* or treb* or tripl*) adj3 (blind* or mask*)).ab,ti.	244444
47	or/20-46 [**study designs]	11009994
48	allergy/	81119
49	antibody/	240916
50	CD4 antigen/	122632
51	exp chemokine/	215047
52	exp communicable disease/	29048
53	cytokine/	239842
54	endocrine system/	180095
55	exp immune system/	2264983
56	exp immunity/	1566062
57	exp immunoglobulin/	525460
58	infection/	395080
59	inflammation/	458324
60	autacoid/	6326
61	interleukin 1/	63814
62	luminescence/	27684
63	monocyte/	115087
64	neutrophil/	143602
65	radioimmunoassay/	85142
66	respiratory burst/	9581
67	"selectin (glycoprotein)"/	181
68	Th1 cell/	37631
69	Th2 cell/	33231
70	tumor necrosis factor/	126974
71	virus/	86679

© 2021 Chow N et al. *JAMA Network Open*.

72	allergy.ab,ti.	112329
73	(anti-allerg* or antiallerg*).ab,ti.	7807
74	"antibod*".ab,ti.	1129774
75	CD4.ab,ti.	213717
76	"chemokine*".ab,ti.	95545
77	"cytokine*".ab,ti.	492200
78	(disease* adj4 (communicable or infectious)).ab,ti.	144693
79	endocrine.ab,ti.	183409
80	(immune or immunity).ab,ti.	973061
81	"immunoglobulin*".ab,ti.	196408
82	immunology.ab,ti.	45580
83	"infection*".ab,ti.	1845658
84	"inflammation*".ab,ti.	630961
85	(Interleukin-1 or Interleukin-2).ab,ti.	67053
86	"luminescent measurement*".ab,ti.	29
87	monocytes.ab,ti.	98125
88	neutrophils.ab,ti.	108852
89	radioimmunoassay.ab,ti.	58760
90	respiratory burst.ab,ti.	6550
91	selectins.ab,ti.	2495
92	substance P.ab,ti.	25443
93	(Th1 or Th2).ab,ti.	75705
94	(TNFalpha or TNF-alpha).ab,ti.	202440
95	Tumor Necrosis Factor-alpha.ab,ti.	85373
96	or/48-95 [**immune_system]	6783955
97	19 and 47 and 96	2291
98	limit 97 to english language	2199
99	limit 98 to (books or chapter or conference abstract or conference paper or "conference review" or editorial or erratum or letter or note or "review")	941
100	98 not 99	1259

CINAHL
CCBC_RR3_Immunesystem_CINAHL
Search run April 15, 2020 in CINAHL Plus with Full Text; 433 results

#	Query	Results
S82	S16 AND S33 AND S80<br>Limiters - English Language; Peer Reviewed<br>Expanders - Apply equivalent subjects<br>Search modes - Boolean/Phrase	433
S81	S16 AND S33 AND S80	490

© 2021 Chow N et al. *JAMA Network Open*.

**S80**	**S34 OR S35 OR S36 OR S37 OR S38 OR S39 OR S40 OR S41 OR S42 OR S43 OR S44 OR S45 OR S46 OR S47 OR S48 OR S49 OR S50 OR S51 OR S52 OR S53 OR S54 OR S55 OR S56 OR S57 OR S58 OR S59 OR S60 OR S61 OR S62 OR S63 OR S64 OR S65 OR S66 OR S67 OR S68 OR S69 OR S70 OR S71 OR S72 OR S73 OR S74 OR S75 OR S76 OR S77 OR S78 OR S79**	**854,487**
**S79**	**Tumor Necrosis Factor-alpha**	**6,767**
**S78**	**TNFalpha or TNF-alpha**	**9,624**
**S77**	**Th1 or Th2**	**2,635**
**S76**	**substance P**	**140,242**
**S75**	**selectins**	**1,813**
**S74**	**respiratory burst**	**183**
**S73**	**radioimmunoassay**	**3,329**
**S72**	**neutrophils**	**14,833**
**S71**	**monocytes**	**8,974**
**S70**	**luminescent measurement***	**636**
**S69**	**Interleukin-1 or Interleukin-2**	**4,406**
**S68**	**inflammation***	**87,052**
**S67**	**infection***	**372,110**
**S66**	**immunology**	**78,867**
**S65**	**immunoglobulin***	**31,445**
**S64**	**immune or immunity**	**77,934**
**S63**	**endocrine**	**18,176**
**S62**	**disease* N4 (communicable or infectious)**	**32,870**
**S61**	**cytokine***	**41,368**
**S60**	**chemokine***	**6,497**
**S59**	**CD4**	**16,615**
**S58**	**biomarker***	**61,248**
**S57**	**antibod***	**81,281**
**S56**	**anti-allerg* or antiallerg***	**556**
**S55**	**allergy**	**20,570**
**S54**	**MH Viruses**	**4,986**
**S53**	**MH Tumor Necrosis Factor**	**19,034**
**S52**	**MH Radioimmunoassay**	**2,262**

© 2021 Chow N et al. *JAMA Network Open*.

**S51**	**MH Neutrophils**	**6,580**
**S50**	**MH Monocytes**	**3,214**
**S49**	**MH Luminescent Measurements**	**636**
**S48**	**MH Interleukins+**	**25,516**
**S47**	**MH Inflammation Mediators**	**6,116**
**S46**	**MH Inflammation**	**38,408**
**S45**	**MH Infection**	**16,455**
**S44**	**MH Immunoglobulins**	**17,617**
**S43**	**MH Immunity**	**15,171**
**S42**	**MH Immune System**	**9,400**
**S41**	**MH Endocrine System**	**1,130**
**S40**	**MH Communicable Diseases+**	**11,828**
**S39**	**MH Cytokines**	**21,892**
**S38**	**MH Chemokines**	**1,843**
**S37**	**MH CD4 Lymphocyte Count**	**6,126**
**S36**	**MH Biological Markers**	**53,963**
**S35**	**MH Antibodies**	**10,923**
**S34**	**MH "Allergy and Immunology"**	**1,703**
**S33**	**S17 OR S18 OR S19 OR S20 OR S21 OR S22 OR S23 OR S24 OR S25 OR S26 OR S27 OR S28 OR S29 OR S30 OR S31 OR S32**	**1,542,090**
**S32**	**(singl* or doubl* or treb* or tripl*) N3 (blind* or mask*)**	**80,244**
**S31**	**retrospective N3 (stud* or design*)**	**292,000**
**S30**	**random* N5 (allocat* or assign* or control* or clinical or patient* or sample* or trial*)**	**373,577**
**S29**	**prospective N3 (stud* or design* or analysis)**	**506,212**
**S28**	**longitudinal* N3 (stud* or design* or analysis)**	**58,131**
**S27**	**control* N3 trial***	**211,800**
**S26**	**cohort***	**220,234**
**S25**	**(followup or follow-up) N3 (stud* or design* or analysis)**	**30,976**
**S24**	**(crossover or cross-over) N3 (study or studies or trial*)**	**13,647**
**S23**	**case N3 (control* or series or report*)**	**253,610**
**S22**	**MH Randomized Controlled Trials**	**118,608**
**S21**	**MH Random Sample+**	**114,961**

© 2021 Chow N et al. *JAMA Network Open*.

**S20**	**PT Clinical Trial**	**110,541**
**S19**	**PT Randomized Controlled Trial**	**130,867**
**S18**	**(MH "Nonexperimental Studies+")**	**796,292**
**S17**	**MH Case-Control Studies**	**0**
**S16**	**S1 OR S2 OR S3 OR S4 OR S5 OR S6 OR S7 OR S8 OR S9 OR S10 OR S11 OR S12 OR S13 OR S14 OR S15**	**24,932**
**S15**	**(therap* or treat* or intervention* or manag*) N3 (manual or manipulat* or mobili?at* or MSK or musculoskeletal)**	**17,015**
**S14**	**(manipulat* or mobili?at*) N4 instrument***	**260**
**S13**	**mobili?at* N3 (spinal or spine or low* back or joint* or lumbar or neck or thoracic or cervical or MSK or musculoskeletal or vertebr*)**	**1,809**
**S12**	**mobili?at* N3 (chiropract* or naprapath* or osteopath* or orthopedic* or orthopaedic*)**	**67**
**S11**	**manipulat* N3 (physiotherap* or physical therap*)**	**275**
**S10**	**manipulat* N3 (instrument assisted or instrument-assisted)**	**19**
**S9**	**manipulat* N3 (spinal or spine or low* back or joint* or lumbar or neck or thoracic or cervical or MSK or musculoskeletal or vertebr*)**	**3,155**
**S8**	**manipulat* N3 (chiropract* or naprapath* or osteopath* or orthopedic* or orthopaedic*)**	**8,018**
**S7**	**HVLA or high velocity low amplitude**	**359**
**S6**	**flexion-distraction or flexion distraction**	**140**
**S5**	**adjust* N3 (chiropract* or spinal or lumbar or cervical or thoracic or instrument* or tool* or electric)**	**1,455**
**S4**	**Activator N2 (method or tool*)**	**107**
**S3**	**MH Manipulation, Osteopathic**	**768**
**S2**	**MH Manipulation, Orthopedic**	**2,444**
**S1**	**MH Manipulation, Chiropractic**	**4,370**

Cochrane Central
CCBC_RR3 _Immunesystem_Central
Search run April 16, 2020 in EBM Reviews - Cochrane Central Register of Controlled Trials February 2020; 237 results

**1**	**Musculoskeletal Manipulations/**	**413**
**2**	**Manipulation, Spinal/**	**353**
**3**	**Manipulation, Chiropractic/**	**124**
**4**	**Manipulation, Orthopedic/**	**245**
**5**	**Manipulation, Osteopathic/**	**128**
**6**	**(Activator adj (method or tool*)).ti,kw,ab.**	**3**

© 2021 Chow N et al. *JAMA Network Open.*

7	(adjust* adj3 (chiropract* or spinal or lumbar or cervical or thoracic or instrument* or tool* or electric)).ti,kw,ab.	353
8	(flexion-distraction or flexion distraction).ti,kw,ab.	26
9	(HVLA or high velocity low amplitude).ti,kw,ab.	192
10	(manipulat* adj3 (chiropract* or naprapath* or osteopath* or orthopedic* or orthopaedic*)).ti,kw,ab.	605
11	(manipulat* adj3 (spinal or spine or low* back or joint* or lumbar or neck or therap* or thoracic or cervical or intervention* or MSK or manage* or musculoskeletal or treat* or vertebr*)).ti,kw,ab.	2734
12	(instrument* adj3 (manual or mobili?at* or manipulat*)).ti,kw,ab.	214
13	(manipulat* adj3 (physiotherap* or physical therap*)).ti,kw,ab.	174
14	(mobili?at* adj3 (chiropract* or naprapath* or osteopath* or orthopedic* or orthopaedic*)).ti,kw,ab.	33
15	(mobili?at* adj3 (spinal or spine or low* back or joint* or lumbar or neck or thoracic or cervical or MSK or musculoskeletal or vertebr* or therap* or treat* or intervention* or manage*)).ti,kw,ab.	1765
16	(mobili?at* adj3 (spinal or spine or low* back or joint* or lumbar or neck or thoracic or cervical or MSK or musculoskeletal or vertebr* or therap* or treat* or intervention* or manage*)).ti,kw,ab.	1765
17	or/1-16 [**intervention]	5422
18	Case-Control Studies/	5045
19	Clinical Trials as Topic/	33229
20	clinical trial.pt.	279746
21	Cohort Studies/	7409
22	controlled clinical trial.pt.	91385
23	Controlled Clinical Trials as Topic/	71
24	Cross-Over Studies/	36488
25	Double Blind Method/	135429
26	Epidemiologic Studies/	41
27	Follow-Up Studies/	58629
28	Longitudinal Studies/	6184
29	Observational Study.pt.	1103
30	Prospective Studies/	90273
31	Random Allocation/	20629
32	randomized controlled trial.pt.	490034
33	exp Randomized Controlled Trial/	131
34	Randomized Controlled Trials as Topic/	5891
35	Retrospective Studies/	8451
36	Single Blind Method/	20296
37	((crossover or cross-over) adj3 (study or studies or trial*)).ti,kw,ab.	67202
38	((followup or follow-up) adj3 (stud* or design* or analysis)).ti,kw,ab.	40817

© 2021 Chow N et al. *JAMA Network Open*.

39	"cohort*".ti,kw,ab.	58835
40	(longitudinal* adj3 (stud* or design* or analysis)).ti,kw,ab.	9248
41	(observational adj3 (stud* or design* or analysis)).ti,kw,ab.	15696
42	(prospective adj3 (stud* or design* or analysis)).ti,kw,ab.	115914
43	(random* adj5 (allocat* or assign* or control* or clinical or patient* or sample* or trial*)).ti,kw,ab.	844623
44	(retrospective adj3 (stud* or design*)).ti,kw,ab.	17848
45	((singl* or doubl* or treb* or tripl*) adj3 (blind* or mask*)).ti,kw,ab.	294302
46	or/18-45 [**study designs]	1203560
47	"Allergy and Immunology"/	4
48	exp Antibodies/	19479
49	Biomarkers/	13725
50	exp CD4 Lymphocyte Count/	2186
51	exp Chemokines, CC/	413
52	exp Communicable Diseases/	1795
53	Cytokines/	3049
54	exp Endocrine System/	4225
55	exp Immune System/	11976
56	exp Immunity/	3151
57	exp Immunoglobulins/	20192
58	exp Infection/	23633
59	Inflammation/	5052
60	nflammation Mediators/	0
61	exp Interleukin-1/	888
62	Luminescent Measurements/	168
63	Monocytes/	758
64	Neutrophils/	1380
65	Radioimmunoassay/	998
66	Respiratory Burst/	121
67	exp Selectins/	709
68	Th1 Cells/	232
69	Th2 Cells/	233
70	exp Tumor Necrosis Factors/	3400
71	exp Viruses/	8960
72	allergy.ti,kw,ab.	10922
73	(anti-allerg* or antiallerg*).ti,kw,ab.	688
74	"antibod*".ti,kw,ab.	34892
75	"biomarker*".ti,kw,ab.	27613
76	CD4.ti,kw,ab.	10756

© 2021 Chow N et al. *JAMA Network Open*.

77	**"chemokine*".ti,kw,ab.**	**1736**
78	**"cytokine*".ti,kw,ab.**	**16410**
79	**(disease* adj4 (communicable or infectious)).ti,kw,ab.**	**4301**
80	**endocrine.ti,kw,ab.**	**10178**
81	**(immune or immunity).ti,kw,ab.**	**33846**
82	**"immunoglobulin*".ti,kw,ab.**	**8203**
83	**immunology.ti,kw,ab.**	**2985**
84	**"immunoglobulin*".ti,kw,ab.**	**8203**
85	**"infection*".ti,kw,ab.**	**94682**
86	**"inflammation*".ti,kw,ab.**	**35877**
87	**(Interleukin-1 or Interleukin-2).ti,kw,ab.**	**3680**
88	**"uminescent measurement*".ti,kw,ab.**	**0**
89	**monocytes.ti,kw,ab.**	**1970**
90	**neutrophils.ti,kw,ab.**	**3266**
91	**radioimmunoassay.ti,kw,ab.**	**2127**
92	**respiratory burst.ti,kw,ab.**	**145**
93	**selectins.ti,kw,ab.**	**40**
94	**(Th1 or Th2).ti,kw,ab.**	**1820**
95	**(TNFalpha or TNF-alpha).ti,kw,ab.**	**8003**
96	**Tumor Necrosis Factor-alpha.ti,kw,ab.**	**4203**
97	**or/47-96 [**immunesystem]**	**264067**
98	**17 and 46 and 97**	**334**
99	**limit 98 to english language**	**237**

© 2021 Chow N et al. *JAMA Network Open*.

eTable 1. Evidence Table for Immune Markers

1st Author, Year	Study design, Participants, Setting, Number (n) enrolled	Interventions, Provider, Number (n) of subjects at baseline	Comparison, Provider, Number (n) of subjects at baseline	Follow-up	Outcomes	Results	Quality of Evidence	Conclusion (notes)[a]
Brennan 1994[1]	RCT; adult patients with LBP; duration >50 days; 45% female<br><br>Chiropractic teaching clinic in Lombard, USA<br><br>(n=201)	SMT: HVLA thrust to T12 to S1 (including SI joints), according to clinical indication by chiropractors in teaching clinic<br><br>11 tx/2 wks<br><br>(n=67)	1. LFP sham tx: delivered to one level of the L/S<br><br>11 tx/2 wks by chiropractors in teaching clinic<br><br>(n=67)<br><br>2. Lecture series: educational lectures on LBP, no physical contact with physician and no exercise recommendations<br><br>(n=67)	i. At 12th visit<br>ii. 2 wks post-tx<br><br>Loss to follow-up: 52/201=26% (unclear distribution across groups)	B lymphocytes<br>T lymphocytes<br>T_H cells<br>T_S cells<br>NK lymphocytes	**<u>At 12th visit</u>**<br><br>1. **<u>SMT vs. LFP sham:</u>**<br>Mean difference at 12th visit in % of lymphocyte:<br>Total T cells: 0.4 (-1.4, 22)<br>T_H cells: 0.1 (-3.1, 3.4)<br>T_S cells: 0.4 (-2.0, 2.8)<br>NK cells: -0.5 (-2.6, 1.6)<br>T_SNK cells: -0.4 (-1.3, 0.5)<br>B cells: -1.5 (-3.4, 0.4)<br><br>2. **<u>SMT vs. lecture series:</u>**<br>Mean difference at 12th visit in % of lymphocyte:<br>Total T cells: 0.5 (-1.6, 2.6)<br>T_H cells: -2.4 (-5.4, 0.6)<br>T_S cells: 1.9 (-0.4, 4.2)<br>NK cells: -0.8 (-3.1, 1.5)<br>T_SNK cells: -0.2 (-1.2, 0.9)<br>B cells: -0.5 (-2.7, 1.7)<br><br>3. **<u>LFP sham vs. lecture series:</u>**<br>Mean difference at 12th visit in % of lymphocyte:<br>Total T cells: -0.1 (-2.1, 1.9)	High (++)	SMT is not associated with lymphocyte levels

© 2021 Chow N et al. *JAMA Network Open*.

						T_H cells: 2.5 (-0.2, 5.2)<br>T_S cells: -1.5 (-3.6, 0.6)<br>NK cells: 0.3 (-1.9, 2.5)<br>T_SNK cells: -0.2 (-1.1, 0.7)<br>B cells: -1 (-3.2, 1.2)<br><br>**<u>2 wks post-tx</u>**<br><br>**4. <u>SMT vs. LFP sham:</u>**<br>Mean difference at 2 wks in % of lymphocyte:<br>Total T cells: 1.2 (-0.6, 3.0)<br>T_H cells: -0.4 (-3.5, 2.7)<br>T_S cells: 0.2 (-2.2, 2.6)<br>NK cells: 0.2 (-1.8, 2.2)<br>T_SNK cells: -0.1 (-1.0, 0.8)<br>B cells: -1.1 (-2.9, 0.7)<br><br>**5. <u>SMT vs. lecture series:</u>**<br>Mean difference at 2 wks in % of lymphocyte:<br>Total T cells: 1.0 (-1.1, 3.1)<br>T_H cells: -2.9 (-5.6, -0.2)<br>T_S cells: 2.0 (-0.3, 4.3)<br>NK cells: 0.1 (-1.9, 2.1)<br>T_SNK cells: 0 (-1.0, 1.0)<br>B cells: -0.6 (-2.6, 1.4)<br><br>6. **<u>LFP sham vs. lecture series</u>**:<br>Mean difference at 2 wks in % of lymphocyte:<br>Total T cells: 0.2 (-1.9, 2.3)<br>T_H cells: 2.5 (-0.4, 5.4)		

© 2021 Chow N et al. *JAMA Network Open*.

						T_S cells: -1.8 (-4.1, 0.5)<br>NK cells:0.1 (-2.1, 2.3)<br>T_SNK cells: -0.1 (-1.0, 0.8)<br>B cells: -0.5 (-2.6, 1.6)<br><br>Each lymphocyte subpopulation (Total T cells, T_H cells, T_S cells, NK cells, T_SNK cells, and B cells) were outcomes in repeated measures ANOVA with primary test of the treatment group by time interaction. None of the treatment by time interactions were significant		
Brennan 1991[2]	RCT; healthy adult 1st year chiropractic students (naïve to SMT); mean age 26.2 (SD 5.5); 32% female<br><br>Chiropractic teaching clinic in Lombard, USA<br><br>(n=99)	SMT:<br>HVLA thrust (posterior to anterior direction) from T1-T6<br><br>One session by chiropractor in teaching clinic<br><br>(n=42)	1. Sham:<br>low-velocity light-force thrust (prone to anterior direction) to selected segment<br><br>One session by chiropractor in teaching clinic<br><br>(n=38)<br><br>2. Soft tissue manipulation:	15 min post-tx<br><br>Loss to follow-up/missing data:<br><br>PMN:<br>SMT: 9.5%<br>Sham: 7.9%<br>Soft tissue:21%<br><br>Substance P:<br>SMT: 25%<br>Sham: 36%	CL response of PMN<br>CL response of monocytes<br>Substance P	**CL response of PMN**<br><br>Pre-post difference in mean peak/sec/cell[b]:<br>SMT (n=38): 2.9<br>Sham (n=35): 0.2<br>Soft tissue (n=15): -0.03<br><br>**CL response of monocytes**<br><br>Pre-post difference in mean peak/sec/cell[c]:<br>SMT (n=38): 0.71<br>Sham (n=42): 0.09<br>Soft tissue (n=19): 0.07<br><br>**Substance P**	Acceptable<br>(+)	SMT is associated with changes in CL response to PMN and monocytes<br><br>(clinical meaning of effect is unknown

© 2021 Chow N et al. *JAMA Network Open*.

			to either right or left gluteal area<br><br>One session by chiropractor in teaching clinic<br><br>(n=19)			Mean difference of change in pg/mL: 11.1. This difference is not significantly different from zero (p-value not reported)<br><br>No data for SP were collected for soft tissue group		SMT is not associated with substance P levels
Teodorczyk-Injeyan 2006[3]	RCT; healthy adult chiropractic students; 56% female<br><br>Chiropractic teaching clinic in Toronto, Canada<br><br>(n=64)	SMT: HVLA thrust (Carver-Bridge) with cavitation in anterior and superior direction to the T/S<br><br>One session by chiropractor in teaching clinic<br><br>(n=24)	1. Sham: HVLA thrust (Carver-Bridge) without cavitation (positioning and line of drive that did not cavitate joint)<br><br>One session by chiropractor in teaching clinic<br><br>(n=20)<br><br>2. Venipuncture control (VC) No tx<br><br>(n=20)	i. 20 min post-tx<br>ii. 2 hrs post-tx<br><br>No loss to follow-up	TNF-α[d]<br>IL-1β[d]<br>Substance P[d]	**<u>20 min post-tx</u>**<br><br>**<u>SMT vs. Sham:</u>**<br>Mean difference in amount of change from baseline (pg/mL):<br><br>TNF-α: 471 (-29.5, 971.5)<br><br>IL-1β: 223 (-205.8, 651.8)<br><br>**<u>SMT vs. VC:</u>**<br>Mean difference in amount of change from baseline (pg/mL):<br><br>TNF-α: 358 (-74.1, 790.1)<br><br>IL-1β: 156 (-256.4, 568.4)<br><br>**<u>Sham vs. VC:</u>**<br>Mean difference in amount of change from baseline (pg/mL):	Acceptable<br>(+)	SMT associated with attenuation of TNF-α and IL-1β production levels at 2 hrs, but not at 20 minutes<br><br>(clinical meaning of effect is unknown)

© 2021 Chow N et al. *JAMA Network Open*.

						TNF-α: 113 (-405.8, 631.8)<br><br>IL-1β: 67 (-352.1, 486.1)<br><br>**<u>2 hrs post-tx</u>**<br><br>**<u>SMT vs. Sham</u>**<u>:</u><br>Mean difference in amount of change from baseline (pg/mL):<br><br>TNF-α: 1091 (497.0, 1685.0)<br><br>IL-1β: -612 (97.3, 1126.7)<br><br>**<u>SMT vs. VC:</u>**<br>Mean difference in amount of change from baseline (pg/mL):<br><br>TNF-α: 800 (249.3, 1350.7)<br><br>IL-1β: 565 (96.3, 1033.7)<br><br>**<u>Sham vs. VC:</u>**<br>Mean difference in amount of change from baseline (pg/mL):<br><br>TNF-α: 291 (-314.9, 896.9)<br><br>IL-1β: 67 (-352.1, 486.1)		

© 2021 Chow N et al. *JAMA Network Open*.

						Substance P findings reported in bar charts: no statistically significant differences between groups at follow-up.<br>Baseline mean values of substance P were 55, 51 and 54 pg/mL for the VC, sham and SMT groups respectively		 SMT-C is not associated with substance P levels
Teodorczyk-Injeyan 2008[4]	RCT; healthy adult chiropractic students; 56% female<br><br>Chiropractic teaching clinic in Toronto, Canada	SMT:<br>HVLA thrust (Carver-Bridge) with cavitation in anterior and superior direction to the T/S	1. Sham: HVLA thrust (Carver-Bridge) without cavitation (positioning and line of drive that did not cavitate joint)	1. 20 min post-tx<br>ii. 2 hrs post-tx<br><br>Loss to follow-up:<br>SMT: 3.3%<br>Sham: 8%<br>VC: 11%	IL-2 induced by SPA[e] or TPA[e]	**<u>20 min post-tx</u>**<br><br><u>IL-2 production induced by SPA[e]:</u><br><br>**<u>SMT vs. Sham:</u>**<br>No significant difference (p=0.436)<br><br>**<u>SMT vs. VC:</u>**	Acceptable<br>(+)	SMT and sham associated with increase in IL-2 levels at 20 min and 2 hrs compared to VC

© 2021 Chow N et al. *JAMA Network Open*.

	(n=82)	One session by chiropractor in teaching clinic<br><br>(n=30)	One session by chiropractor in teaching clinic<br><br>(n=25)<br><br>2. Venipuncture control (VC)<br>No tx<br><br>(n=27)			Significant difference (p=0.000)<br><br>**Sham vs. VC:**<br>Significant difference (p=0.006)<br><br>IL-2 production induced by TPA+ calcium ionophore[e]:<br><br>No significant difference between groups (p=0.085)<br><br>**2 hrs post-tx**<br><br>IL-2 production induced by SPA[e]:<br><br>**SMT vs. Sham:**<br>No significant difference (p=0.737)<br><br>**SMT vs. VC:**<br>Significant difference (p=0.001)<br><br>**Sham vs. VC:**<br>Significant difference (p=0.003)<br><br>IL-2 production induced by TPA+ calcium ionophore[e]:<br><br>No significant difference between all groups (p=0.802)		(clinical meaning of effect is unknown)<br><br>No difference between SMT and sham

© 2021 Chow N et al. *JAMA Network Open*.

Teodorczyk-Injeyan 2010[5]	RCT; healthy adult chiropractic students; 56% female<br><br>Chiropractic teaching clinic in Toronto, Canada<br><br>(n=82)	SMT:<br>HVLA thrust (Carver-Bridge) with cavitation in anterior and superior direction to the T/S<br><br>One session by chiropractor in teaching clinic<br><br>(n=30)	1. Sham: HVLA thrust (Carver-Bridge) without cavitation (positioning and line of drive that did not cavitate joint)<br><br>One session by chiropractor in teaching clinic<br><br>(n=25)<br><br>2. Venipuncture control (VC)<br>No tx<br><br>(n=27)	i. 20 min post-txii. 2 hrs post-tx<br><br>Loss to follow-up:<br>SMT: 10%<br>Sham: 0%<br>VC: 18.5%	IgG<br>IgM<br>B (CD19) lymphocytes<br>T (CD 3) lymphocytes	**<u>PWM-induced antibody production</u>**<br>The mean production of both IgG and IgM in cultures from VC, sham and SMT subjects were unchanged<br><br>**<u>IL-2-induced antibody production</u>**<br><br>**<u>IgG</u>**<br>Repeated measures ANOVA of the transformed data demonstrated a statistically significant group by time interaction effect (F=2.8, p=0.03). At 20 min post-treatment, the mean production of IgG in the SMT group was significantly higher than that in the VC and sham groups.[d] At 2 hr post-treatment, the production of IgG in cultures from both SMT and sham was significantly elevated compared to VC.[d]<br><br>**<u>IgM</u>**<br>Repeated measures ANOVA of the transformed data demonstrated a statistically significant group by time	Acceptable<br>(+)	SMT associated with IL-2-induced augmentation of IgG and IgM levels[f]<br><br>(clinical meaning of effect is unknown)

© 2021 Chow N et al. *JAMA Network Open*.

						interaction effect (F=2.7, p=0.04). At 2 hr posttreatment the mean level of IgM synthesis in the SMT group was significantly elevated compared with the VC group.[e]<br><br>**Phenotypic analysis**<br><br>**SMT vs. Sham**:<br>Mean difference in change from baseline (%)[e]<br><br>B (CD19) lymphocytes: 0.6<br>T (CD3) lymphocytes: -6.0<br><br>**SMT vs. VC:**<br>Mean difference in change from baseline (%)[e]<br><br>B (CD19) lymphocytes: 1.0<br>T (CD3) lymphocytes: -4.0<br><br>**Sham vs. VC:**<br>Mean difference in change from baseline (%)[e]<br><br>B (CD19) lymphocytes: 0.4<br>T (CD3) lymphocytes: 2.0		SMT is not associated with B and T lymphocyte levels

© 2021 Chow N et al. *JAMA Network Open*.

Abbreviations: CL chemiluminescence, cpm counts per minute, CRP C-reactive protein, C/S cervical spine, HIV human immunodeficiency virus, hrs hours, HVLA high velocity low amplitude, IgG immunoglobulin G, IgM immunoglobulin M, IL interleukin, LBP low back pain, LFP low force, high velocity low amplitude procedure, LPS lipopolysaccharide, L/S lumbar spine, min minutes, MT manual treatment, MVMA moderate velocity moderate amplitude, NK natural killer cells, ODI Oswestry Disability Index, OMT osteopathic manipulative treatment, PHA phytohaemagglutinin, PMN Polymorphonuclear neutrophils, PWM pokeweed mitogen, RCT randomized controlled trial, SI sacroiliac joint, SMT spinal manipulative therapy, SMT spinal manipulative therapy with cavitation, US ultrasound, T_H T-helper cells, TNF-α tumor necrosis factor α, T_S T-suppressor cells, T/S thoracic spine, Tx treatment, UK United Kingdom, USA United States of America, VAS visual analogue scale, w/o without, w/ with, wks weeks, yo years old

[a]Red: no effect of SMT; green: effect of SMT; yellow: results depend on processing of cells.
[b]Derived from regression models in Table 1, using a pre value of 3.0. Note that intercepts in the model are not significantly different, but the slope for SMT is significantly higher ($p<.005$) than the slope for either sham or soft tissue.
[c]Derived from regression models in Table 2, using a pre value of 1.75. Note that intercepts in the model are not significantly different, but the slope for SMT is significantly higher ($p<.005$) than the slope for either sham or soft tissue.
[d]SI conversion factors: To convert TNF-α from pg/mL to nM/L, multiply values by 5.85×10^{-5}. To convert IL-1β from pg/mL to nM/L, multiply values by 5.85×10^{-5}. To convert substance P from pg/mL to pmol/ L, multiply values by 0.742.
[e]Unable to calculate mean difference (95% CI) due to lack of data reported.
[f]While a point estimate of the magnitude of the effect can be determined, a confidence interval cannot with the data as reported.

© 2021 Chow N et al. *JAMA Network Open*.

eTable 2. Evidence Table for Endocrine and Other Physiological Markers

1st Author, Year	Study design, Participants, Setting, Number (n) enrolled	Interventions, Provider, Number (n) of subjects at baseline	Comparison, Provider, Number (n) of subjects at baseline	Follow-up	Outcome Measures	Results	Quality of Evidence	Conclusion (notes)[a]
Mackawan 2007[6]	RCT; patients with chronic LBP (>12 wks), 20-60 yo, 61% female<br><br>Physical therapy department in Khon Kaen, Thailand<br><br>(n=67)	TTM<br>Deep massage with prolonged pressure (10 min) on the muscles along with passive stretching<br><br>One session by physical therapist<br><br>(n=35)	Joint mobilization<br>Grade 2 mobilization, 5 min/set, 2 sets/level at lumbar spinous process of L2–L5<br><br>One session by physical therapist<br><br>(n=32)	5 min post-tx<br><br>No loss to follow-up	Substance P[b]	Mean difference (95% CI) in change from pre- to post-tx (pg/mL): 1.2 (-21.6, 24.1)	Acceptable (+)	SMT is not associated with substance P levels
Puhl 2012[7]	RCT; healthy adults, 20-45 yo, 47% female<br><br>Chiropractic teaching clinic in Toronto, Canada	SMT:<br>HVLA thrust, either 1) combination type adjustment (hypothenar transverse push) for segments T1 to T3,	Sham:<br>HVLA thrust, either combination or Carver-Bridge without cavitation	i. immediately post-tx<br>ii. 15 min post-tx<br><br>Loss to follow-up:<br>SMT: 5.3%<br>Sham: 14.3%	Norepinephrine[b]<br>Epinephrine[b]	**<u>Immediately post-tx</u>**<br><br>**<u>SMT vs. Sham</u>**:<br>Mean difference in amount of change from baseline (nM):<br><br>Norepinephrine: -0.06 (-0.26, 0.38)[c]<br>Epinephrine: 0.05 (-	Acceptable (+)	SMT is not associated with norepinephrine and epinephrine levels

© 2021 Chow N et al. *JAMA Network Open*.

	(n=56)	or 2) Carver-Bridge type adjustment (bilateral hypothenar push) for segments T4 to T6<br><br>One session by chiropractor in teaching clinic<br><br>(n=26)	One session by chiropractor in teaching clinic<br><br>(n=30)			0.39, 0.29[c]<br><br>**<u>15 min post-tx</u>**<br><br>**<u>SMT vs. Sham</u>**:<br>Mean difference in amount of change from baseline (nM):<br><br>Norepinephrine: 0.01 (-0.07, 0.05)[c]<br>Epinephrine: 0.01 (-0.07, 0.05)[c]		
Sampath 2017[8]	RCT; healthy men, 18-45 yo<br><br>Recruited through advertising<br><br>Physical therapy department in Dunedin, New Zealand<br><br>(n=24)	SMT<br>HVLA thrust to T/S through the participant's upper extremity and thorax upon expiration<br><br>One session by physical therapist<br><br>(n=12)	Sham tx identical to SMT but therapist did not place a fixating hand against the thoracic spine, no thrust<br><br>One session by physical therapist<br><br>(n=12)	i. 5 min post-tx<br>ii. 30 min post-tx<br>iii. 6 hrs post-tx<br><br>No loss to follow-up	Salivary cortisol[b]<br>Testosterone[b]<br>T/C ratio[b]<br>O_2Hb[b]<br>HRV[b]	**<u>5 min post-tx</u>**<br><br><u>Mean difference (95% CI) in (pg/m)</u><br><br>Cortisol: -0.4 (-0.6, -0.12)[d]<br>Testosterone: 10.1 (-43.5, 63.8)<br>T/C ratio: 0.04 (-0.05, 0.13)<br>O_2Hb: 0.89 (13.9, 15.6)<br>HRV: -0.11 (-0.47, 0.25)<br><br>**<u>30 min post-tx</u>**<br><br><u>Mean difference (95% CI) in (pg/m)</u><br><br>Cortisol: -0.20 (-0.46, 0.05)	Acceptable (+)	SMT associated with changes in cortisol levels at 5 minutes only<br><br>(clinical meaning of effect is unknown)<br><br>SMT is not associated with levels of testosterone, T/C ratio, O_2Hb, and HRV

© 2021 Chow N et al. *JAMA Network Open*.

						Testosterone: -6.29 (-60, 47.5)<br>T/C ratio: 0.00 (-0.09, 0.09)<br>O_2Hb: 15.2 (-30.5, 60.9)<br>HRV: 0.01 (-0.47, 0.25)<br><br>**<u>6 hrs post-tx</u>**<br><br><u>Mean difference (95% CI) in (pg/m)</u><br><br>Cortisol: 0.23 (-0.06, 0.53)<br>Testosterone: -12.9 (-47.5, 21.8)<br>T/C ratio: -0.09 (-0.16, -0.04)<br><br>O^2Hb and HRV data not reported for 6 hrs post-tx		

Abbreviations: C/S cervical spine, HRV heart rate variability, HVLA high velocity low amplitude, LBP low back pain, min minutes, NO nitric oxide, O_2Hb oxyhemoglobin, RCT randomized controlled trial, SD standard deviation, SMT spinal manipulative therapy, T/C ratio testosterone/cortisol ratio, T/S thoracic spine, TTM traditional Thai massage, Tx treatment, USA United States of America, w/o without, w/ with, wks weeks, yo years old

[a]Red: no effect of SMT; green: effect of SMT; yellow: results depend on processing of cells.
[b]SI conversion factors: To convert substance P from pg/mL to pmol/ L, multiply values by 0.742.To convert norepinephrine from pg/mL to pmol/L, multiple values by 5.911. To convert epinephrine from pg/mL to pmol/L, multiple values by 5.459. To convert cortisol from µg/dL to nmol/L, multiple values by 27.588. To convert testosterone from ng/dL to nmol/L, multiple values by 0.0347.
[c]We report difference in mean change scores and 95% CI using the reported mean change scores and standard errors from Table 2 and 3. Note that in two instances in Table 2, the reported mean change scores cannot be reproduced taking differences in means. There is clearly an error in reporting, but we are unable to identify which values are in error.
[d]The reported results cannot be used because they are statistically incorrect. The reported p value was 0.005. There was likely a typo in the reporting of results; therefore, we imputed the 95% CI.

© 2021 Chow N et al. *JAMA Network Open*.

eReferences

1. Brennan PC, Graham MA, Triano JJ, Hondras MA, Anderson RJ. Lymphocyte profiles in patients with chronic low back pain enrolled in a clinical trial. *J Manipulative Physiol Ther.* 1994;17(4):219-227.
2. Brennan PC, Kokjohn K, Kaltinger CJ, et al. Enhanced phagocytic cell respiratory burst induced by spinal manipulation: potential role of substance P. *J Manipulative Physiol Ther.* 1991;14(7):399-408.
3. Teodorczyk-Injeyan JA, Injeyan HS, Ruegg R. Spinal manipulative therapy reduces inflammatory cytokines but not substance P production in normal subjects. *J Manipulative Physiol Ther.* 2006;29(1):14-21.
4. Teodorczyk-Injeyan JA, Injeyan HS, McGregor M, Harris GM, Ruegg R. Enhancement of in vitro interleukin-2 production in normal subjects following a single spinal manipulative treatment. *Chiropr Osteopat.* 2008;16:5.
5. Teodorczyk-Injeyan JA, McGregor M, Ruegg R, Injeyan HS. Interleukin 2-regulated in vitro antibody production following a single spinal manipulative treatment in normal subjects. *Chiropr Osteopat.* 2010;18:26.
6. Mackawan S, Eungpinichpong W, Pantumethakul R, Chatchawan U, Hunsawong T, Arayawichanon P. Effects of traditional Thai massage versus joint mobilization on substance P and pain perception in patients with non-specific low back pain. *Journal of Bodywork and Movement Therapies.* 2007;11(1):9-16.
7. Puhl AA, Injeyan HS. Short-term effects of manipulation to the upper thoracic spine of asymptomatic subjects on plasma concentrations of epinephrine and norepinephrine-a randomized and controlled observational study. *J Manipulative Physiol Ther.* 2012;35(3):209-215.
8. Sampath KK, Botnmark E, Mani R, et al. Neuroendocrine response following a thoracic spinal manipulation in healthy men. *J Orthop Sports Phys Ther.* 2017;47(9):617-627.
9. Tricco AC, Antony J, Zarin W, et al. A scoping review of rapid review methods. *BMC Med.* 2015;13:224.
10. Tricco AC, Langlois E, Straus S. *Rapid Reviews to Strengthen Health Policy and Systems: A Practice Guide.* Geneva: World Health Organization; 2017.
11. Varker T, Forbes D, Dell L, et al. Rapid evidence assessment: increasing the transparency of an emerging methodology. *J Eval Clin Pract.* 2015;21(6):1199-1204.
12. Featherstone RM, Dryden DM, Foisy M, et al. Advancing knowledge of rapid reviews: An analysis of results, conclusions and recommendations from published review articles examining rapid reviews. *Syst Rev.* 2015;4:50.
13. Corso M, Cancelliere C, Mior S, Kumar V, Smith A, Côté P. The clinical utility of routine spinal radiographs by chiropractors: a rapid review of the literature. *Chiropr Man Therap.* 2020;28(1):33.
14. Corso M, Cancelliere C, Mior S, Taylor-Vaisey A, Côté P. The safety of spinal manipulative therapy in children under 10 years: a rapid review. *Chiropr Man Therap.* 2020;28(1):12.
15. Moher D, Liberati A, Tetzlaff J, Altman DG, The Prisma Group. Preferred Reporting Items for Systematic Reviews and Meta-Analyses: The PRISMA Statement. *PLOS Medicine.* 2009;6(7):e1000097.
16. Rubinstein SM, Terwee CB, Assendelft WJ, de Boer MR, van Tulder MW. Spinal manipulative therapy for acute low-back pain. *Cochrane Database Syst Rev.* 2012;2012(9):Cd008880.
17. Maitland G, Hengeveld E, Banks K, English K. *Maitland's Vertebral Manipulation.* 7th ed. Toronto: Elsevier Butterworth Heinemann; 2005.
18. Dagenais S, Haldeman S. *Evidence-based management of low back pain.* Amsterdam, Netherlands: Elsevier; 2012.

© 2021 Chow N et al. *JAMA Network Open.*

19. Biomarkers Definition Working Group. Biomarkers and surrogate endpoints: Preferred definitions and conceptual framework. *Clin Pharmacol Ther.* 2001;69(3):89-95.
20. Scottish Intercollegiate Guidelines Network. Methodology: Checklists. https://www.sign.ac.uk/what-we-do/methodology/checklists/. Published 2013. Accessed August 23, 2020.
21. Campbell CM, Gilron I, Doshi T, Raja S. Designing and conducting proof-of-concept chronic pain analgesic clinical trials. *Pain Rep.* 2019;4(3):e697.
22. Campbell M, McKenzie JE, Sowden A, et al. Synthesis without meta-analysis (SWiM) in systematic reviews: reporting guideline. *BMJ.* 2020;368:l6890.
23. Hewitt CE, Kumaravel B, Dumville JC, Torgerson DJ. Assessing the impact of attrition in randomized controlled trials. *J Clin Epidemiol.* 2010;63(11):1264-1270.
24. Kjaergard LL, Villumsen J, Gluud C. Reported methodologic quality and discrepancies between large and small randomized trials in meta-analyses. *Ann Intern Med.* 2001;135(11):982-989.
25. Pildal J, Hróbjartsson A, Jørgensen KJ, Hilden J, Altman DG, Gøtzsche PC. Impact of allocation concealment on conclusions drawn from meta-analyses of randomized trials. *Int J Epidemiol.* 2007;36(4):847-857.
26. Trowman R, Dumville JC, Torgerson DJ, Cranny G. The impact of trial baseline imbalances should be considered in systematic reviews: A methodological case study. *J Clin Epidemiol.* 2007;60(12):1229-1233.
27. Wood L, Egger M, Gluud LL, et al. Empirical evidence of bias in treatment effect estimates in controlled trials with different interventions and outcomes: Meta-epidemiological study. *Bmj.* 2008;336(7644):601-605.

© 2021 Chow N et al. *JAMA Network Open*.
